# Disseminated Peritoneal Nematode Infestation Causing Adhesive Small Bowel Obstruction and Bowel Gangrene Following Perforated Appendicitis: A Case Report

**DOI:** 10.7759/cureus.110568

**Published:** 2026-06-09

**Authors:** Anvitha Reddy Gouni, Satya Bala, Vineeth Vishwanath, Ramachandran P Iyyappan, Saravanan Sanniyasi

**Affiliations:** 1 General Surgery, Sri Ramachandra Institute of Higher Education and Research, Chennai, IND

**Keywords:** case report, parasitic appendicitis, peritoneal nematode infestation, rare case presentation, small bowel obstruction

## Abstract

Disseminated peritoneal nematode infestation is a rare extra-intestinal manifestation of ascariasis, typically occurring following bowel perforation. We report a case of a male in his 60s presenting with features of acute small bowel obstruction, including abdominal pain, distension, obstipation, and bilious vomiting, with a history of perforated appendicitis managed surgically six months prior.

Contrast-enhanced computed tomography suggested small bowel obstruction. Intraoperative findings revealed dense adhesions, gangrenous ileum, and multiple nodular lesions over the small bowel, mesentery, and omentum. Resection of the gangrenous segment with primary anastomosis was performed. Histopathological examination confirmed nematode infestation with associated granulomatous inflammation. The findings suggest transperitoneal dissemination following prior appendicular perforation. This case highlights a rare presentation of ascariasis mimicking granulomatous disease and emphasizes the importance of considering parasitic etiologies in atypical intra-abdominal pathology, particularly in endemic regions.

## Introduction

Ascariasis, caused by the parasitic roundworm *Ascaris lumbricoides*, remains one of the most common helminthic infections worldwide and continues to be recognized among the World Health Organization’s neglected tropical diseases [1]. The disease predominantly affects populations in tropical and subtropical regions with poor sanitation and hygiene, where fecal-oral transmission remains the primary mode of spread [1,2]. Although most infections are asymptomatic or confined to the intestinal lumen, heavy worm infestation may lead to serious gastrointestinal complications, including intestinal obstruction, volvulus, perforation, appendicitis, biliary ascariasis, bowel ischemia, and, in severe cases, gangrene [3,4]. In endemic regions, ascariasis remains an underrecognized cause of acute abdomen.

Extra-intestinal disseminated peritoneal nematode infestation is rare and is usually associated with disruption of the intestinal wall, allowing parasitic material to spread into the peritoneal cavity [5]. Similar cases of peritoneal dissemination following bowel perforation have been reported in the literature [6]. Ascariasis has also been implicated in uncommon surgical presentations such as gastric perforation [7], appendicitis [8], and appendiceal infestation in children [9]. Granulomatous peritonitis secondary to Ascaris infestation has likewise been described, highlighting the ability of parasitic material to induce chronic inflammatory reactions within the peritoneal cavity [10-14].

Intestinal obstruction remains one of the most well-recognized complications of parasitic infestation, particularly when associated with inflammatory adhesions or bowel compromise [15]. Disseminated peritoneal nematode infestation is an exceptionally rare extra-intestinal manifestation of ascariasis. To date, the literature consists predominantly of isolated case reports and small case series describing granulomatous peritonitis or peritoneal dissemination following intestinal perforation. We report a rare case of disseminated peritoneal nematode infestation presenting as adhesive small bowel obstruction with intestinal gangrene in a patient with a previous history of perforated appendicitis, highlighting the diagnostic challenges associated with extra-intestinal helminthic disease.

## Case presentation

In December 2023, a male in his sixth decade of life with no known comorbidities presented with colicky abdominal pain, abdominal distension, and obstipation for two days, along with two episodes of bilious vomiting over the preceding day. He had undergone an open appendicectomy six months earlier for perforated appendicitis. The postoperative period was complicated by a superficial surgical site infection, which was managed conservatively with antibiotics. He had no other history of abdominal surgery.

On presentation, the patient was hemodynamically stable, alert, and well-oriented. Abdominal examination revealed generalized distension and tenderness without signs of generalized peritonitis. The appendicectomy scar was healthy, with no cough impulse. Bowel sounds were absent on auscultation. Routine hematological investigations showed elevated white count, and biochemical investigations were within normal limits.

Based on the clinical history and examination findings, the differential diagnoses included postoperative adhesive small bowel obstruction, which was considered the most likely diagnosis given the recent history of abdominal surgery; obstructed incisional hernia, although no clinically evident hernia was identified; small bowel neoplasm causing mechanical obstruction; intestinal tuberculosis with adhesive or stricturing disease; and, less commonly, inflammatory or parasitic granulomatous lesions resulting in bowel obstruction.

Given the clinical suspicion of acute intestinal obstruction, contrast-enhanced computed tomography (CECT) of the abdomen was performed. Imaging demonstrated an abrupt transition point in the distal ileum, with proximal dilatation of the jejunal and ileal loops (maximum diameter: 3.2 cm) and collapse of the distal bowel loops, consistent with acute mechanical small bowel obstruction.

The patient was taken up for emergency diagnostic laparoscopy. Intraoperatively, hemorrhagic ascites, dilated small bowel loops, dense interloop adhesions, and an adhesive band causing distal ileal obstruction were identified. A segment of ileum appeared gangrenous, and multiple firm nodules were noted over the bowel serosa, mesentery, and omentum. Owing to extensive adhesions, bowel distension, and the presence of gangrenous bowel requiring definitive management, the procedure was converted to a midline laparotomy.

**Figure 1:**
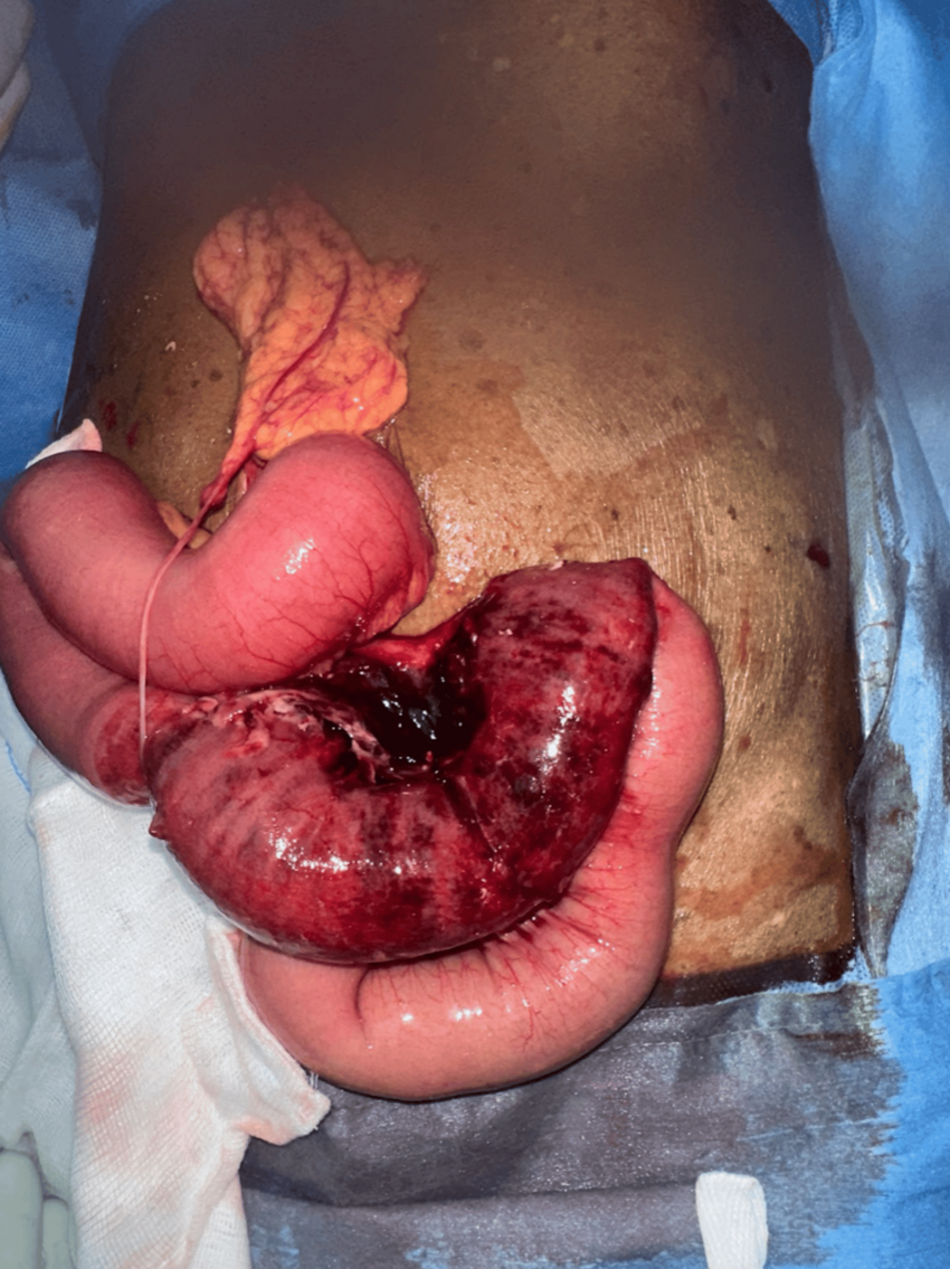
The gangrenous segment of the small bowel with the adhesion band causing the obstruction.

**Figure 2:**
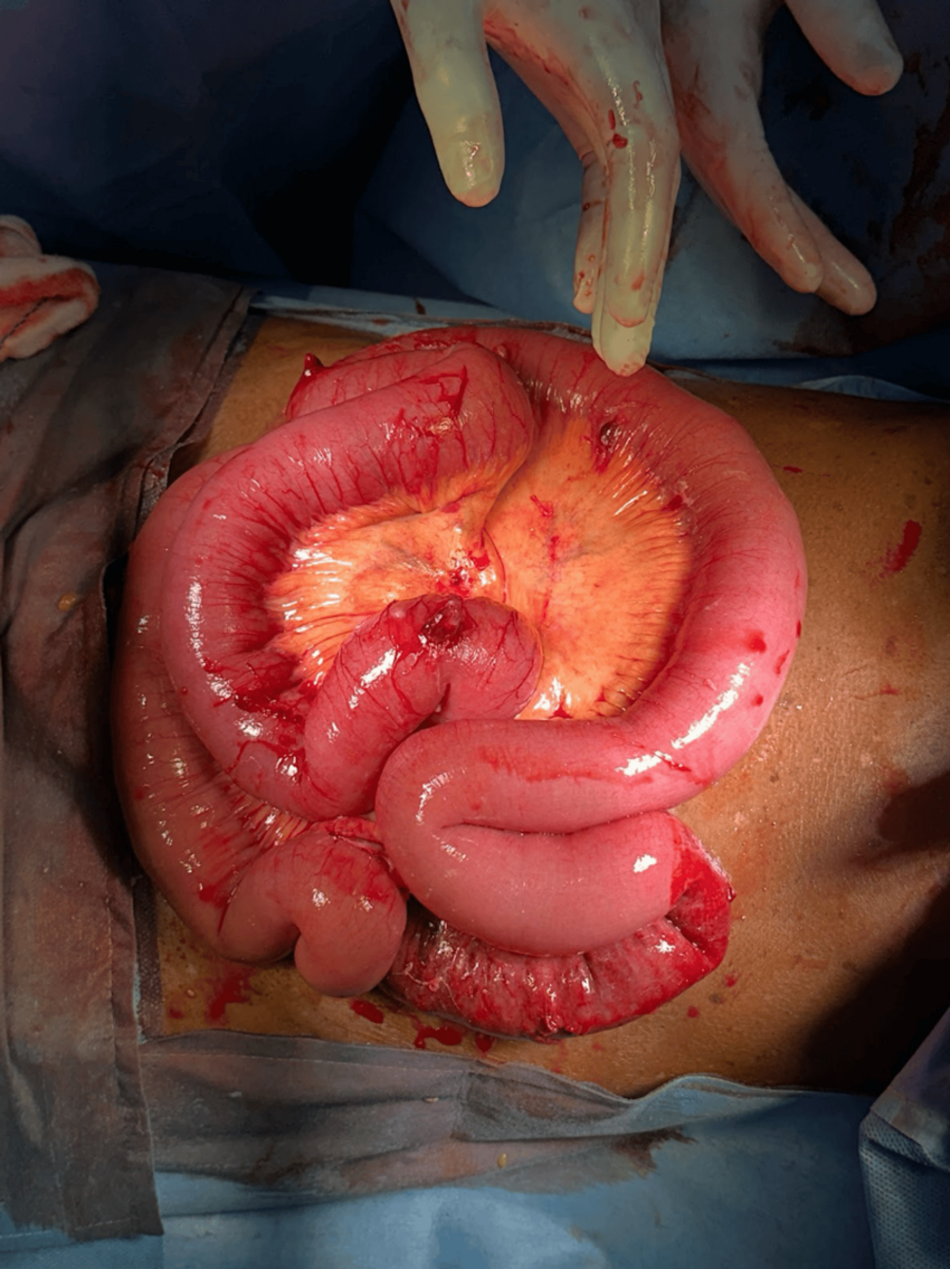
Dilated small bowel loops with multiple nodules in the small bowel wall, mesentery, and omentum.

**Figure 3:**
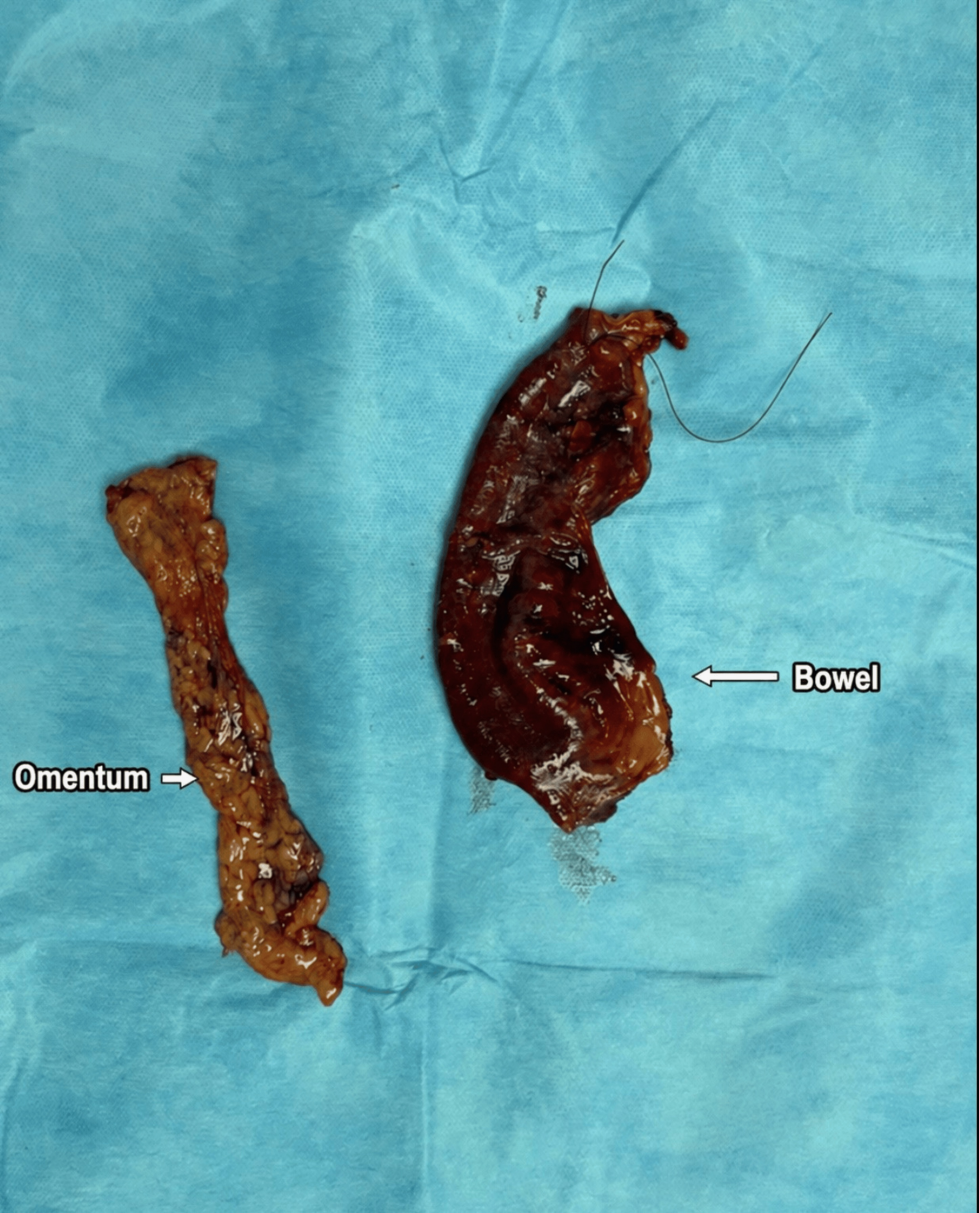
Resected small bowel loop, along with the biopsied segment of omentum.

Following adhesiolysis and systematic bowel examination, a gangrenous ileal segment approximately 60 cm proximal to the ileocecal junction was identified. Segmental resection of the gangrenous bowel was performed, followed by primary stapled end-to-end anastomosis using GIA staplers (Dublin, Ireland: Medtronic). Multiple biopsies were obtained from the nodules involving the bowel serosa, mesentery, and omentum (Figures 1-3).

Postoperative recovery was uneventful. The patient resumed oral intake on postoperative day two and passed stool by day four. Oral feeds were initiated on postoperative day two and were well tolerated. The patient passed flatus on postoperative day two and stool on postoperative day four.

**Figure 4:**
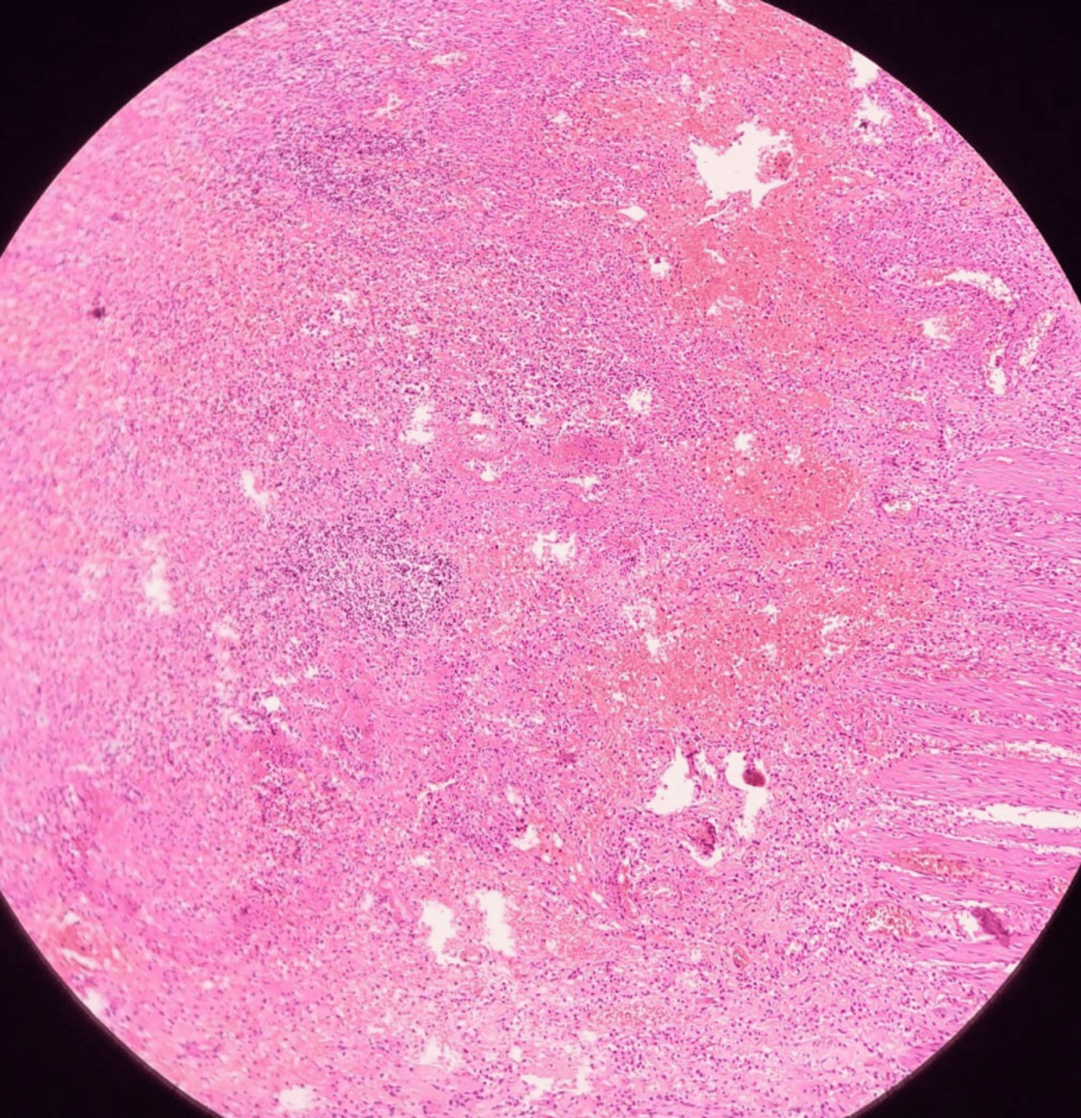
Cross section of the gangrenous bowel segment showing areas of hemorrhage and inflammation (H&E stain, 100× magnification).

**Figure 5:**
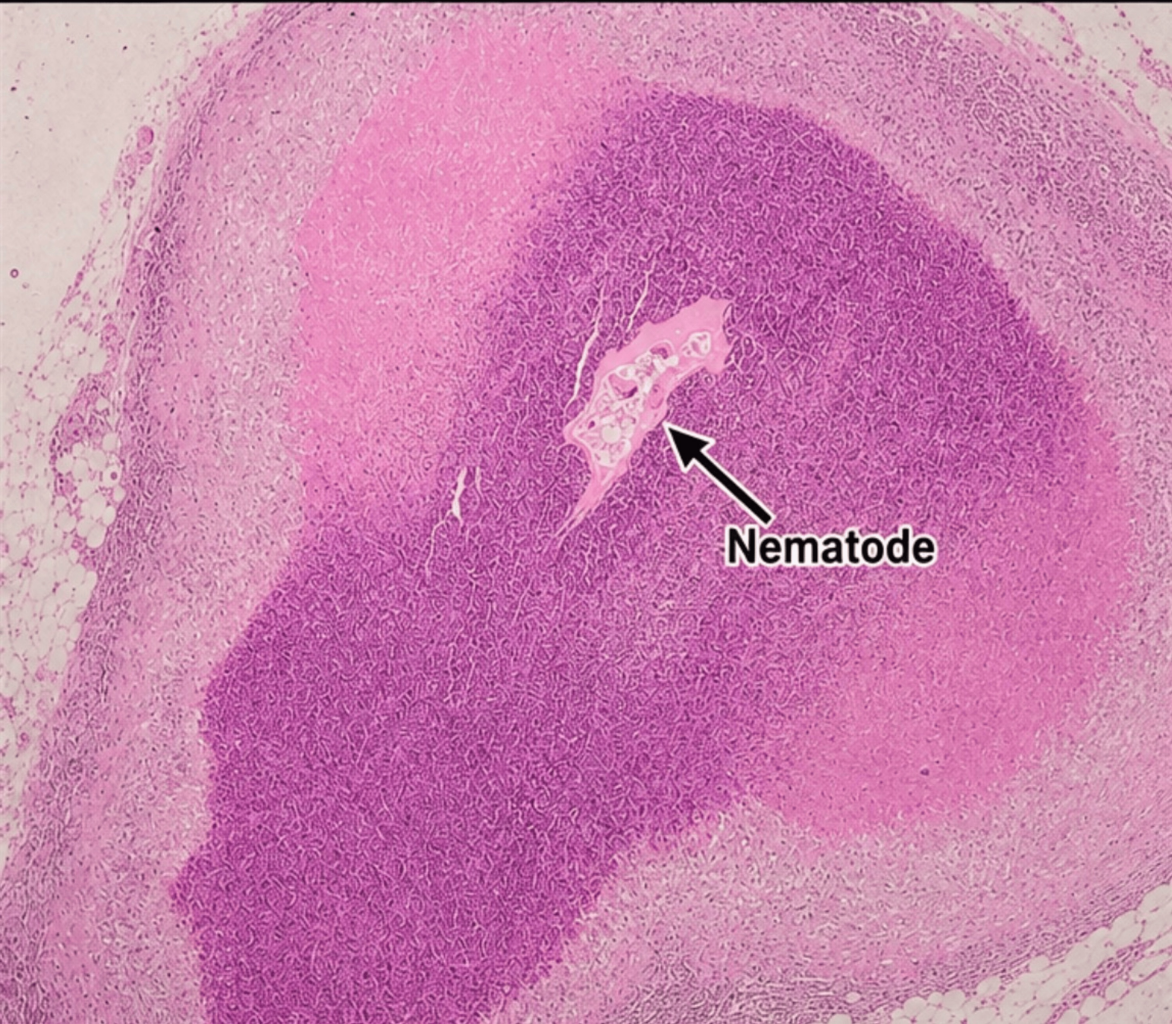
Histopathological image of a cross-section of an omental nodule demonstrating parasitic structures morphologically consistent with nematode infestation associated with granulomatous inflammation (H&E stain, 40× magnification).

**Figure 6:**
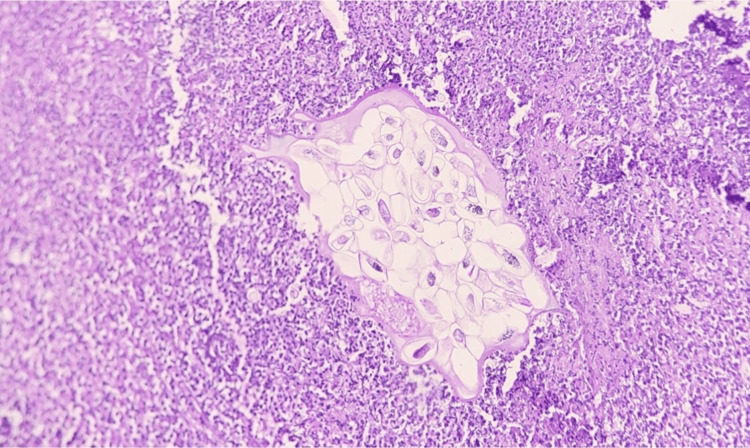
Histopathological section of omental nodule showing cross-sections of nematodes (H&E stain, 400× magnification).

Histopathological examination of the resected bowel demonstrated acute suppurative inflammation with gangrenous changes. Biopsies from the omental and mesenteric nodules revealed encapsulated necrotic lesions containing parasitic structures morphologically consistent with the phylum Nematoda, accompanied by granulomatous inflammation. Species identification could not be established. Postoperative stool examination was negative for ova and parasites. The patient was treated with a single oral dose of albendazole 400 mg. At subsequent follow-up, he remained asymptomatic, with no recurrence of symptoms or further complications (Figures 4-6).

## Discussion

Ascariasis remains an important public health problem in tropical and developing countries, particularly in regions with poor sanitation and inadequate deworming practices [1,2]. *Ascaris lumbricoides* is one of the most common soil-transmitted helminths worldwide and is usually acquired through the fecal-oral route [1]. Most infections remain asymptomatic or confined to the intestinal lumen. However, complications may occur depending on the worm burden, host immune response, and associated bowel pathology. Intestinal obstruction is the most common surgical complication, especially in children, whereas bowel ischemia, volvulus, perforation, appendicitis, and peritonitis are relatively rare but potentially life-threatening manifestations [3,4,15].

Peritoneal dissemination of nematodes represents a rare extra-intestinal complication of helminthic infestation and has been described in association with ascariasis in previous reports [5,10]. Although the exact pathophysiology remains unclear, disruption of the bowel wall or intestinal perforation may allow ova, larvae, or parasitic material to migrate into the peritoneal cavity [5]. In the present case, the patient had undergone surgery for perforated appendicitis six months earlier, which may represent one possible predisposing factor for transperitoneal dissemination of parasitic material with subsequent granulomatous inflammatory reaction within the peritoneum, mesentery, and omentum. However, a direct causal relationship cannot be definitively established. Several cases of granulomatous peritonitis secondary to *Ascaris lumbricoides* have been reported in the literature, suggesting that parasitic material within the peritoneal cavity can trigger chronic inflammatory and fibrotic reactions [10,13,14].

The inflammatory response associated with disseminated nematode infestation may lead to dense adhesions involving bowel loops, mesentery, and omentum, thereby predisposing patients to adhesive small bowel obstruction [12]. In severe cases, delayed presentation or vascular compromise may result in bowel ischemia, necrosis, and gangrene, as observed in this patient. The presence of disseminated peritoneal nodules associated with adhesive small bowel obstruction and gangrenous bowel is extremely rare.

Another notable feature of this case was the presence of multiple hard nodules over the bowel serosa, mesentery, and omentum, which mimicked other pathological conditions, such as peritoneal tuberculosis, peritoneal carcinomatosis, Crohn’s disease, fungal granulomas, and chronic inflammatory disorders [10,13,14]. Typical imaging findings described in intestinal ascariasis include echogenic tubular structures with central anechoic lines on ultrasonography and linear or tubular filling defects on computed tomography [15]. However, these findings may be absent in disseminated extra-intestinal disease, making preoperative diagnosis difficult. Stool examination may also remain negative despite extensive tissue involvement, as seen in this patient. Definitive diagnosis is therefore usually established intraoperatively and confirmed by histopathological examination, demonstrating parasitic structures associated with granulomatous inflammation [10,13].

In the present case, peritoneal tuberculosis and peritoneal carcinomatosis were considered important differential diagnoses because of the presence of multiple peritoneal and omental nodules associated with adhesive bowel obstruction. However, histopathological examination did not demonstrate malignant cells, caseating granulomas, or features suggestive of tuberculosis. The identification of parasitic structures associated with granulomatous inflammatory reaction favored disseminated nematode infestation as the most likely diagnosis.

Management depends on the clinical presentation and associated complications. Uncomplicated intestinal ascariasis can usually be treated conservatively with anti-helminthic medications such as albendazole or mebendazole [3]. Surgical intervention becomes necessary in patients presenting with intestinal obstruction, bowel ischemia, perforation, peritonitis, or gangrene [4,15]. In the present case, the patient underwent diagnostic laparoscopy, which was converted to exploratory laparotomy, allowing adhesiolysis, resection of the gangrenous bowel segment, and tissue diagnosis. Postoperatively, the patient was treated with albendazole 400 mg orally as a single dose and had an uneventful recovery.

This case highlights several important clinical considerations. First, parasitic etiologies should be included in the differential diagnosis of atypical intra-abdominal granulomatous lesions, particularly in endemic regions [11]. Second, disseminated peritoneal nematode infestation may clinically and intraoperatively mimic malignancy, tuberculosis, or chronic granulomatous disorders, making histopathological examination essential for definitive diagnosis [10,13,14]. Third, previous bowel perforation may represent one of the mechanisms facilitating extra-intestinal dissemination of nematode infestation, although further molecular and pathological studies are required to establish causality [5,12].

The limitations of this report include the inability to identify the exact nematode species and the lack of molecular analysis. Although histopathological findings were morphologically consistent with nematode infestation, definitive species identification could not be established. Despite these limitations, the findings provide strong evidence suggestive of disseminated parasitic infestation involving the peritoneal cavity.

## Conclusions

This case highlights that disseminated peritoneal nematode infestation can present as small bowel obstruction and mimic other granulomatous intra-abdominal diseases. Histopathology is essential when imaging and operative findings are nonspecific.
